# Feasibility of Sustainable Asphalt Concrete Materials Utilizing Waste Plastic Aggregate, Epoxy Resin, and Magnesium-Based Additive

**DOI:** 10.3390/polym15153293

**Published:** 2023-08-03

**Authors:** Sang-Yum Lee, Tri Ho Minh Le

**Affiliations:** 1 Faculty of Civil Engineering, Induk University, 12 Choansan-ro Nowon-gu, Seoul 01878, Republic of Korea; yummy10041004@gmail.com; 2 Faculty of Civil Engineering, Nguyen Tat Thanh University, 300A Nguyen Tat Thanh Street, District 4, Ho Chi Minh City 70000, Vietnam

**Keywords:** sustainability, asphalt concrete, by-product, waste plastic aggregate, epoxy resin

## Abstract

This research addresses the urgent need for sustainable and durable asphalt mixtures by quantitatively investigating the effects of incorporating waste plastic aggregate (WPA) and magnesium-based additives. This study explores WPA content levels of 3%, 5%, and 7% wt of aggregate in combination with a fixed 3% wt epoxy resin content to the asphalt binder, supplemented with the 1.5% wt magnesium-based additive. The novelty of this research lies in its comprehensive analysis of various performance parameters, including deformation strength, indirect tensile strength (ITS), rut depth, and dynamic stability, to assess the impact of WPA, epoxy resin, and the magnesium-based additive on asphalt mixture properties. The results demonstrate significant improvements in key performance aspects with increasing WPA content. The WPA mixtures exhibit enhanced deformation strength, with values of 4.01, 3.7, and 3.32 MPa for 3, 5, and 7% wt WPA content, respectively, compared to the control mixture. Furthermore, the inclusion of WPA and epoxy resin, along with the magnesium-based additive, contributes to improved adhesion, cohesion, and resistance to stripping damage. Notably, the 7% wt WPA mixture showcases exceptional performance, characterized by a final rut depth of 2.66 mm and a dynamic stability of 7519 passes per millimeter, highlighting its superior rutting resistance and load-bearing capacity. This study also reveals the influence of WPA content on ITS and stiffness properties, with the 5% wt WPA mixture achieving an optimal balance between strength and stiffness. Overall, this research highlights the potential of incorporating WPA, epoxy resin, and magnesium-based additives in asphalt mixtures to enhance their performance and durability. By utilizing plastic waste materials and optimizing their combination with epoxy reinforcement, along with the innovative use of magnesium-based additive, the findings contribute to the development of sustainable infrastructure materials and pave the way for further advancements in the field.

## 1. Introduction

The global infrastructure sector is facing increasing demands for sustainable and durable materials that can effectively address environmental concerns while maintaining high-performance standards [1]. Among the critical components of infrastructure construction, asphalt concrete plays a pivotal role in providing durable and resilient road surfaces [2]. Traditional asphalt mixtures primarily consist of aggregates, asphalt binders, and mineral fillers [3]. However, the environmental impact associated with the extraction of natural aggregates and the disposal of waste materials has prompted researchers to explore innovative approaches for developing sustainable asphalt concrete materials [4].

In recent years, there has been growing interest in incorporating waste materials into asphalt mixtures to reduce environmental burdens and enhance the performance characteristics of the pavement [5]. The utilization of plastic in asphalt has garnered significant attention, with a multitude of studies investigating its potential as an asphalt modifier [6]. Both the dry method, where shredded plastic is blended with aggregate and bitumen, and the wet method, involving the pre-coating of plastic with bitumen before mixing with aggregate, have been extensively explored. In the context of the provided statement, bitumen refers to a sticky, black, and highly viscous form of petroleum commonly used as a binder in road construction and asphalt applications. It serves as a vital component in both the dry and wet methods of incorporating plastic into road materials [7]. Notably, studies by researchers such as Rui Xiao et al. [8] have demonstrated the benefits of incorporating plastic as an asphalt modifier, highlighting its capacity to enhance various performance aspects of asphalt mixtures [8]. These innovative approaches show promise in addressing sustainability concerns and improving the overall resilience of asphalt pavements, making them a crucial area of interest in the field of pavement materials research [9]. Therefore, this research aims to contribute to this growing body of knowledge by exploring the incorporation of waste plastic aggregate (WPA) in asphalt mixtures, along with the addition of epoxy resin and a magnesium-based additive to further enhance the performance of Warm Plastic-Coated Mixture (WPCM) for sustainable and high-performance asphalt pavements.

WPA is one such material that holds significant potential as a sustainable alternative to asphalt concrete [10]. Waste plastics, such as polyethylene and polypropylene, are abundantly available and present a valuable opportunity for recycling and reuse [11]. Utilizing WPA in asphalt mixtures not only diverts plastic waste from landfills but also offers the potential to improve pavement performance through enhanced mechanical properties and resistance to deformation [12].

In the field of asphalt concrete, recent studies have explored the incorporation of WPA as a partial replacement of natural aggregates [6]. Researchers have investigated the effects of different WPA content levels on the mechanical properties and performance of asphalt mixtures. Xu et al. (2021) conducted a study on asphalt mixtures containing WPA and found that the addition of WPA improved the rutting resistance and moisture susceptibility of the mixtures [13]. They found that WPA could be a viable alternative to traditional aggregates in asphalt mixtures. Similarly, in the realm of concrete, researchers have explored the use of WPA as a replacement for coarse aggregates [14]. Studies have shown that incorporating WPA in concrete mixtures can improve the flexural and compressive strength of the material. Abeysinghe et al. (2021) investigated the mechanical properties of concrete incorporating WPA and reported that the inclusion of WPA led to an increase in strength and a reduction in the density of the concrete [15]. They suggested that WPA could be a suitable option for concrete applications. Pavement systems have also been the focus of research involving WPA. Yao et al. (2022) studied the effects of adding WPA to the base course of pavement structures and found that the inclusion of WPA improved the strength and deformation resistance of the pavement [16]. They concluded that WPA could enhance the performance of pavement systems, especially in terms of load-bearing capacity and durability.

Furthermore, the use of WPA in construction materials offers environmental benefits by diverting plastic waste from landfills and reducing the consumption of natural resources [17]. This aligns with the principles of sustainable development and circular economy. In addition to WPA, the use of additives or modifiers in asphalt mixtures has gained considerable attention for enhancing material properties and performance. Epoxy resin, known for its adhesive properties and strength, has been widely investigated as a reinforcing agent in asphalt concrete [18]. The combination of WPA and epoxy resin has the potential to further enhance the cohesion between plastic particles and the asphalt binder, leading to improved load-bearing capacity and resistance to deformation [19]. The application of epoxy resin in hot mix asphalt (HMA) offers significant benefits for enhancing the performance and durability of the mixture [20]. The epoxy resin acts as a reinforcing agent, improving the cohesion and adhesion between asphalt binder and aggregate particles. It enhances the bonding strength, resulting in improved load-bearing capacity and resistance to stripping [21]. By incorporating epoxy resin into the HMA, the mixture exhibits enhanced adhesion and cohesion properties, leading to increased durability and reduced susceptibility to cracking and rutting [22]. The use of epoxy resin in HMA represents a promising approach to developing sustainable and high-performance asphalt pavements with improved mechanical properties and extended service life [18].

A limitation of current findings in the application of WPA in HMA is the lack of research that combines WPA with epoxy resin in HMA mixtures. The synergistic effects of these two additives in enhancing adhesion, cohesion, and load-bearing capacity remain relatively unexplored. Furthermore, there is limited research on the utilization of magnesium as an additive in WPA-based HMA. Magnesium offers benefits such as improved rutting resistance, increased stiffness, and enhanced moisture resistance [23]. Further studies are needed to investigate the combined effects of epoxy resin and magnesium in WPA-based HMA, optimizing their ratios and dosage for improved asphalt performance and sustainability. Understanding the influence of WPA and epoxy resin on key performance indicators is crucial for optimizing the composition of sustainable asphalt mixtures. The findings of this research can contribute to the development of guidelines and recommendations for utilizing WPA and epoxy resin in asphalt concrete construction. Additionally, this study aims to provide insights into the potential of magnesium-based additives in further enhancing the properties and performance of asphalt mixtures. By bridging the gap between sustainable material development and asphalt concrete engineering, this research seeks to pave the way for more environmentally friendly and resilient infrastructure systems. The results obtained will not only contribute to the body of knowledge but also serve as a valuable resource for practitioners and policymakers in their efforts to promote sustainable practices in the construction industry.

This research aims to contribute to the development of sustainable asphalt concrete materials by investigating the quantitative effects of incorporating WPA and epoxy resin. The study focuses on the WPA content levels of 3%, 5%, and 7% wt of aggregate in combination with a fixed 3% wt epoxy resin content to the asphalt binder. The novelty of this research lies in its comprehensive analysis of various performance parameters, including density, air voids, deformation strength, indirect tensile strength (ITS), rut depth, dynamic stability, and deformation strength ratio (S_D_R), to assess the impact of WPA and epoxy resin on asphalt mixture properties.

## 2. Materials and Methods

### 2.1. Materials

The materials used in this study were sourced from reputable suppliers. The asphalt binder and epoxy resins were obtained from the Korean SK petroleum company, known for their high-quality products. The magnesium additive was sourced from a reliable Korea Import Company in Seoul, South Korea [18,19]. The aggregate used in this study was obtained from a local asphalt plant, ensuring proximity and adherence to regional specifications. To maintain the integrity of the materials, proper storage conditions were observed. The asphalt binder and epoxy resins were stored in sealed containers at controlled temperatures to prevent degradation or contamination. The magnesium additive was also stored in a dry and secure environment to maintain its quality. Prior to their use in the experimental mixtures, the materials underwent a pre-treatment process. The aggregate was carefully screened and washed to remove any impurities or deleterious materials. The asphalt binder and epoxy resins were tested for consistency and viscosity to ensure their suitability for the mixing process. These measures were taken to ensure the materials’ quality and reliability in this research study.

#### 2.1.1. Asphalt

In this research, the use of PG 64-22 asphalt played a pivotal role. PG 64-22 asphalt, characterized by its specific properties, was selected as the fundamental binder for the experimental investigation conducted in this study. The choice of PG 64-22 asphalt was based on its desirable characteristics, such as viscosity, penetration, and softening point, as outlined in Table 1. This particular asphalt grade was deemed suitable for this research due to its known performance and established specifications. By utilizing PG 64-22 asphalt as the base binder, the researchers aimed to evaluate and enhance the performance of asphalt mixtures incorporating WPA and magnesium additive.

Table 1 provides a comprehensive overview of the properties of the PG64-22 asphalt binder used in the study. The measured values of various test items are compared to their respective specifications. The penetration value at 25 °C is found to be 75.0 dmm, exceeding the specified range. The flashpoint is determined to be 339.0 °C, satisfying the requirement of being greater than 260 °C. The softening point is measured at 45.8 °C, and the penetration ratio is determined to be 64.1%, both meeting the specified criteria. The rotational viscosity at 120 °C is measured to be 928 mm^2^/s. The DSR and RTFO values indicate good rheological properties of the asphalt binder. Furthermore, the BBR stiffness at −12 °C is measured at 208 MPa, below the specified limit, while the M-value is 0.27, meeting the requirement. These results demonstrate the specific characteristics and properties of the PG64-22 asphalt binder utilized in this research. Additionally, Table 2 presents the overview of the SK epoxy resin used in this research.

#### 2.1.2. Mineral Aggregates

This research incorporated various types of natural aggregates as crucial components in the experimental investigation. As shown in Table 3, coarse aggregates consisting of granite with maximum dimensions of 25, 20, and 13 mm were specifically selected based on their suitability for meeting the research objectives in accordance with ASTM C127 [29]. These coarse granite aggregates played a significant role in determining the mechanical properties of the asphalt mixtures. As for the fine aggregate, granite crushed sand, also known as screenings, with a particle size of 5 mm or less, was employed. This fine aggregate played a vital role in the overall composition of the asphalt mixtures, contributing to their workability and performance. In addition, limestone powder was introduced as a filling material, serving specific purposes and adding to the overall characteristics of the asphalt mixtures. The aggregate selection and utilization in this research were carefully considered to ensure the desired outcomes.

#### 2.1.3. Waste Plastic Aggregate (WPA)

In this research, the utilization of WPA played a significant role in the experimental investigation. WPA was obtained through the recycling of waste plastic and was employed as an artificial aggregate in the manufacturing process of asphalt mixtures. To clarify, the WPA used in this study was obtained through the recycling of waste plastic materials, primarily sourced from post-consumer plastic waste. The waste plastic materials underwent a collection process and were then carefully cleaned to remove any contaminants. Subsequently, the plastics, including polyethylene (PE), polypropylene (PP), and polystyrene (PS), were subjected to shredding to form small particles. These particles were then heated and melted to create the WPA. The WPA was further processed through sieving and sorting to achieve a uniform size distribution suitable for incorporation into the asphalt mixtures.

**Figure 1:**
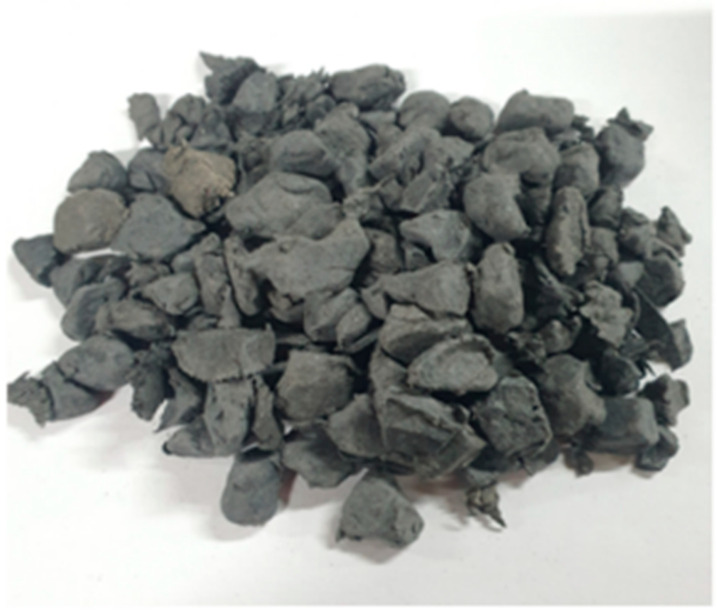
Illustrations of WPCM 5–13 mm.

The objective was to replace a portion of the natural coarse and fine aggregates with WPA. In accordance with ASTM C127, The WPCM was produced with the addition of magnesium as an additive. It was manufactured through extrusion molding at high temperatures, resulting in particle sizes ranging from 5 to 13 mm [29]. The specific compositions and manufacturing methods were selected to explore the potential of WPA in improving the properties and performance of the asphalt mixtures in this research. Table 4 presents the general properties of WPA used in this study, and Figure 1 illustrates the WPA applied in this research. In Table 4, the term “solid content” refers to the portion of the waste plastic material that is non-volatile and remains in solid form after the melting process. The solid content represents the actual WPA that is mixed with the asphalt mixture. The remaining percentage (64.9%) corresponds to the volatile components and gases that are released during the melting process. These volatile components are not included in the final WPA used in the asphalt mixture.

The specific additive used in this research is magnesium powder. It has a particle size range of less than 200 mesh. The specific gravity of the magnesium powder is 1.75, and the bulk density is 315 kg/m^3^ in accordance with AASHTO T27 [30]. The chemical composition of the magnesium powder is predominantly magnesium (Mg), with a purity level exceeding 99.5%. These properties are important in understanding the characteristics and potential effects of the additives in the context of the asphalt mixtures in this research.

To prepare the WPCM, waste plastic was combined with a specific weight percentage of magnesium using the extrusion molding process at a high temperature. The exact amount of magnesium added to the WPCM was carefully controlled to achieve the desired enhancement in stiffness and rutting resistance of the asphalt mixture. The specific weight percentage of magnesium used in the WPCM preparation was 1.5% wt of the total mixture.

The WPA used in this research exhibits a fineness modulus of 6.5, reflecting its particle size distribution. With a bulk density of 0.5, it possesses a relatively low mass per unit volume. The specific gravity under oven-dry conditions is 1.3, indicating its density compared to the density of water. Additionally, the aggregate has a solid content of 36.1%, representing the proportion of solid material present within it. These properties provide valuable insights into the physical characteristics of the WPA, enabling a better understanding of its behavior and potential impact on the asphalt mixtures investigated in this research.

### 2.2. Experimental Methods

#### 2.2.1. Aggregate Property Test

To evaluate the properties of the aggregates used in this study, a series of experiments were carried out. These experiments involved various tests to assess the aggregates’ characteristics. Particle size analysis, conducted following the KS F 2502 standard [30], provided information on the particle size distribution. Density and water absorption measurements were performed to determine the aggregates’ density and water absorption capacity. Additionally, the wear rate of the aggregates was determined using the KS F 2525 standard [31].

#### 2.2.2. Mixture Design

The asphalt concrete mix design employed in this research aimed to achieve the desired properties and performance of the asphalt mixtures. The design process involved carefully selecting and proportioning the constituent materials to meet specific criteria. The aggregate gradation was determined based on the desired particle size distribution, ensuring adequate interlocking and compactness of the mixture. The asphalt binder—in this case, the PG64-22 binder—was selected for its suitable properties and compatibility with the aggregate. The aggregate-to-binder ratio was carefully determined to achieve the desired strength, durability, and workability of the mixture. Additionally, various additives, including magnesium, were incorporated to enhance specific aspects of the mixture’s performance.

The mixing process involved the addition of an additional blend rather than any specific graded aggregate. Voidage and other volume parameters were carefully considered during the mixture design process to determine the optimal amount of asphalt binder for each mixture, aiming to achieve the desired performance characteristics. The revised manuscript will provide clearer explanations to enhance understanding of the mixture design process and the incorporation of WPA in the WPCM.

Table 5 provides information on the gradation and mix design using WPA in different percentages for a specific layer called the base layer. The control mix represents the mix without any WPA content. In this case, for the control mix, no WPA was included (indicated by a hyphen, “-”). The WPCM mix design includes WPA content, with percentages of 3%, 5%, and 7% wt (indicated by the check symbol, “○”). This information allows for a comparison of the mix characteristics and performance based on the WPA content in the base layer. Incorporating 3%, 5%, and 7% wt of WPA was chosen based on preliminary investigations and optimization studies, demonstrating the most promising balance between performance enhancement and cost-effectiveness.

#### 2.2.3. Mixing Process

The mixing process of Hot Mix Asphalt (HMA) incorporating WPA, epoxy resins, and magnesium followed a precise procedure to achieve effective dispersion and integration of these additives. Initially, the aggregate materials were heated in a drum mixer to a specified temperature of approximately 150–160 °C. Following heating, the WPA, which was obtained by recycling waste plastic materials, was added and thoroughly mixed with the aggregate for 2–3 min to ensure uniform distribution. Next, the predetermined amount of epoxy resins was introduced and mixed for an additional 3–5 min to properly coat the WPA particles. The WPA had been previously processed by extrusion molding at a high temperature with the addition of magnesium (1.5% wt of the total mix) to form Waste Plastic Coated Magnesium (WPCM), which enhanced stiffness and rutting resistance. The WPCM was then incorporated into the mixture, and the mixing process continued for about 1–2 min to facilitate thorough blending. Throughout the entire process, strict control of temperature and duration ensured optimal bonding and homogeneity [19].

Notably, the asphalt mixtures used in this study underwent careful manufacturing conditions. The WPA did not undergo heating, while the aggregate was exposed to a temperature of 165 °C for 4 h. The asphalt itself was heated to 160 °C for 2 h and short-term aging was conducted at temperatures ranging from 155 to 160 °C for 1 h. These controlled conditions were essential to ensure proper processing and the attainment of desired asphalt mixture characteristics, showcasing the potential of the resulting HMA with WPA, epoxy resins, and magnesium as a sustainable and high-performance solution for asphalt pavements [18].

Furthermore, the mix design criteria for the asphalt mixtures were established based on the guidelines provided by MOLIT (Ministry of Land, Infrastructure, and Transport) in 2017 [32]. For the BB-2 layer, specific parameters were set to maintain the desired quality and performance. These included a target of 75 gyrations, air voids ranging from 4% to 6%, a minimum VMA (voids in mineral aggregate) of 12.0%, VFA (voids filled with asphalt) within the range of 60% to 75%, a minimum SD (stiffness modulus) of 2.7 MPa, a minimum ITS (indirect tensile strength) of 0.6 MPa, and a toughness of at least 6.0 kN·mm [33]. These criteria serve as benchmarks to ensure that the asphalt mixtures meet the specified requirements and deliver the desired performance for the BB-2 layer.

#### 2.2.4. Curing Conditions

In this study, the curing process for the epoxy resin and WPA composite was carefully determined to achieve the desired bonding and mechanical properties in the asphalt mixtures. The selected curing process involved curing the epoxy–WPA composite at a temperature of 80 °C for a duration of 2 h, ensuring proper cross-linking and adhesion. These parameters were adjusted and fine-tuned based on the specific properties of the epoxy resin and the characteristics of the WPA. The curing process played a critical role in enhancing the cohesion and load-bearing capacity of the asphalt mixtures, contributing to their improved performance and durability [34]. The details of the curing regime will be included in the revised manuscript to provide a comprehensive understanding of its impact on the final properties of the epoxy–WPA composite. This information will enhance the reproducibility and clarity of our study, addressing the Reviewer’s valuable feedback regarding the importance of the curing process in epoxy composites.

#### 2.2.5. Volume Characteristic Evaluation

In order to assess the volume characteristics of the asphalt mixtures, several measurements were conducted. These measurements encompass density, air voids, VMA (voids in mineral aggregate), VFA (voids filled with asphalt), and theoretical maximum density (Gmm). To obtain these measurements, specimens with a diameter of 100 mm were produced using the determined optimal asphalt content and subjected to a total of 70 tests. This comprehensive evaluation allowed for a thorough understanding of the volume characteristics of the asphalt mixtures, providing valuable insights into their performance and suitability for the intended application [35].

#### 2.2.6. Durability Test Items and Contents

Table 6 presents the performance tests conducted on the asphalt mixtures to evaluate their characteristics. The tests included deformation strength (SD) measured at 60 °C with a loading rate of 30 mm/min, indirect tensile strength (ITS) assessed at 25 °C under dry conditions with a loading rate of 50.8 mm/min, wheel tracking (WT) performed at 60 °C with a loading rate of 42 passes per minute, tensile strength ratio (TSR) measured at both 60 and 25 °C under wet conditions for 24 h with a loading rate of 50.8 mm/min, and deformation strength ratio (S_D_R) evaluated at both 60 °C and 25 °C under wet conditions for 48 h with a loading rate of 30 mm/min. These tests were conducted following the respective standards (MOLIT 2017 [33], KS F 2382 [36], KS F 2374 [36] and KS F 2398 [37]) to provide valuable insights into the mechanical properties and performance of the asphalt mixtures under various conditions.

In this research, the mechanical properties of the asphalt concrete mixture were evaluated using the indirect tensile strength (ITS) test according to ASTM D6931 [36]. The test involved the diametrical loading of specimens in a Universal Testing Machine (UTM) to determine the maximum tensile strength and Tensile Strength Ratio (TSR) under both dry and wet conditions. The specimens underwent an aging process with exposure to high temperature and air, followed by water immersion. The resulting TSR ratio values at different temperatures were compared to performance requirements to assess the mixture’s resistance to deformation.

To assess the stiffness and viscoelastic behavior, the dynamic modulus test was conducted following AASHTO TP 62 [38]. Using a Universal Testing Machine (UTM) equipped with a dynamic modulus apparatus, specimens were tested at various temperatures and frequencies. The complex modulus (E*) and phase angle (δ) were measured to construct a master curve of E* as a function of temperature, employing the Time–Temperature Superposition (TTS) principle. The master curve provided a comprehensive view of the mixture’s stiffness over a wide temperature and loading frequency range, enabling evaluation of the effectiveness of the proposed polymer-modified asphalt–concrete mixture.

For evaluating rutting resistance, the Hamburg Wheel Tracking (HWT) test was performed according to AASHTO T324 [38]. Specimens conditioned at 60 °C were subjected to a specified number of cycles using a loaded wheel with a 700 mm diameter. Rut depth measurements were taken to assess the resistance to permanent deformation. The settlement amount was compared to the performance requirement of not exceeding 20 mm after 20,000 cycles.

**Table 6:** Performance test.

Test Item	Temp. (°C)	Condition	Loading	Note
Deformation strength (S_D_)	60		30 mm/min	MOLIT 2017 [32,33] (Figure 2a)
Indirect tensile strength (ITS)	25	Dry	50.8 mm/min	KS F 2382 [36] (Figure 2b)
Wheel tracking (WT)	60	//	42 pass/min	AAHSTO 324 [38] (Figure 2c)
Tensile strength ratio (TSR)	60, 25	Wet 60 °C 24 h	50.8 mm/min	KS F 2398 [37]
Deformation strength ratio (S_D_R)	60, 25	Wet 60 °C 48 h	30 mm/min	-

**Figure 2:**
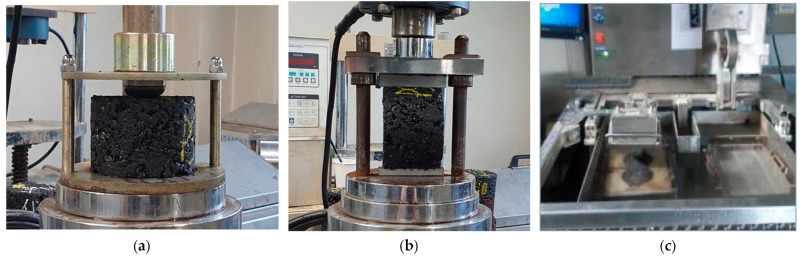
(**a**) Testing device: deformation strength (S_D_) test setting; (**b**) indirect tensile strength (ITS) test setting; (**c**) wheel tracking (WT) test setting.

## 3. Results and Discussion

### 3.1. Mixture Design Results

**Figure 3:**
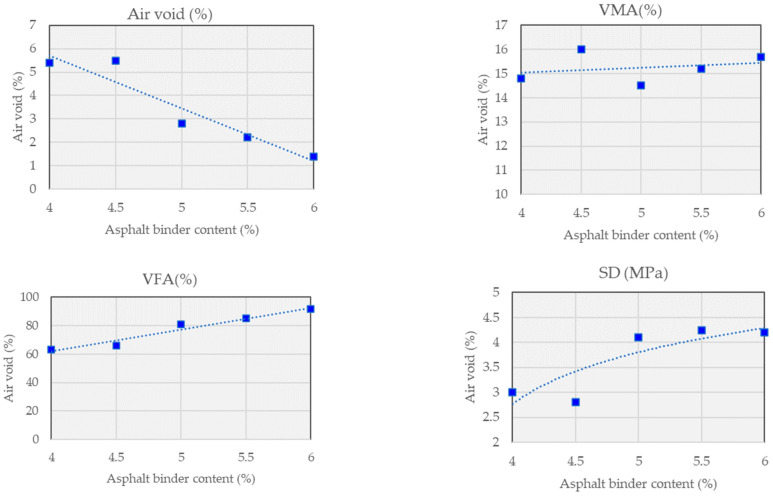
Mix design results of control mix.

**Figure 4:**
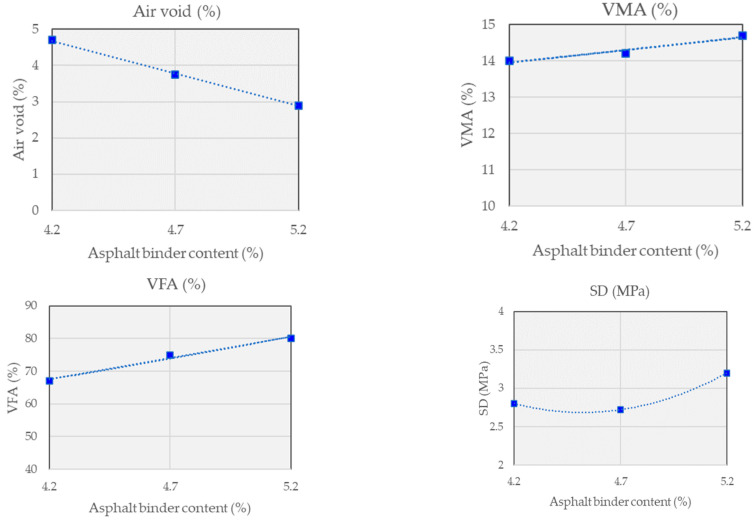
Mix design results of WPCM mix.

Figure 3 and Figure 4 and Table 7 provide information on different asphalt mixtures used in this study, along with their corresponding characteristics. The mixtures are categorized into two types: Control and WPCM. The Control mixture follows the BB-2 gradation. Each row represents a specific asphalt content percentage ranging from 4.0% to 6.0% wt. The test results display the density, air voids percentage, VMA (voids in mineral aggregate) percentage, and VFA (voids filled with asphalt) percentage for each asphalt content level.

For the Control mixture, as the asphalt content increases from 4.0% to 6.0% wt, the density of the mixture shows a slight increase, ranging from 2.389 to 2.419. Simultaneously, the air voids percentage decreases gradually from 5.4% to 1.4%. The VMA percentage remains relatively consistent, ranging from 14.6% to 15.6%, while the VFA percentage significantly increases from 63.4% to 91.3%.

Similarly, for the WPCM mixture with the BB-2 gradation, the asphalt content varies from 4.2% to 5.2%. The density decreases slightly from 2.305 to 2.317 as the asphalt content increases. The air voids percentage decreases from 4.7% to 2.9%. The VMA percentage ranges from 14.1% to 14.6%, while the VFA percentage increases from 67.1% to 80.5%. Overall, these findings provide a comparative overview of the asphalt mixtures’ characteristics, highlighting how changes in asphalt content affect density, air voids, and the distribution of voids within the mixture.

Table 8 presents the Optimum Asphalt Content (OAC) for each asphalt mixture, determined through the mix design process. The OAC represents the optimal amount of asphalt binder needed to achieve the desired performance and characteristics of the asphalt mix. In this table, two mixtures are compared: the control mixture and the WPCM mixture. Both mixtures are classified as BB-2 based on their gradation. The OAC for the control mixture is determined to be 4.2% wt, indicating that 4.2% wt of the total weight of the mixture should consist of asphalt binder. Similarly, the WPCM mixture also has an OAC of 4.2% wt.

### 3.2. Deformation Strength (SD)

**Figure 5:**
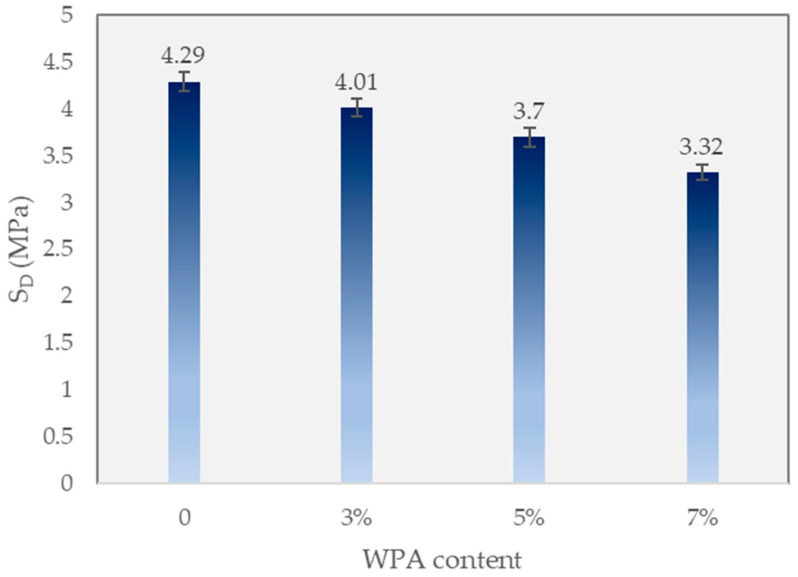
Deformation strength.

Figure 5 illustrates the deformation strength (SD) of different asphalt mixtures with varying percentages of WPA. It is important to note that the WPA mixtures were further enhanced by the inclusion of 3% wt epoxy resin in the asphalt binder, reinforcing the asphalt matrix. Comparing the control mixture with no WPA content, it had a deformation strength of 4.29 MPa. As the WPA content increased in the WPCM mixtures, a slight decrease in deformation strength was observed. The WPCM mixture with 3% wt WPA and epoxy reinforcement exhibited a deformation strength of 4.01 MPa. Further increasing the WPA content to 5% and 7% wt resulted in deformation strengths of 3.7 MPa and 3.32 MPa, respectively. The presence of epoxy resin in the WPCM mixtures helps enhance the bond between the WPA and the asphalt binder, contributing to improved cohesion and load-bearing capacity. Although the deformation strength slightly decreases with increasing WPA content, the addition of epoxy resin helps mitigate any potential negative effects and maintains a satisfactory level of deformation strength. This suggests that the combination of WPA with epoxy reinforcement can be a promising approach for incorporating recycled materials into asphalt mixtures while still ensuring adequate performance and durability.

### 3.3. Strain Strength Ratio (S_D_R)

**Figure 6:**
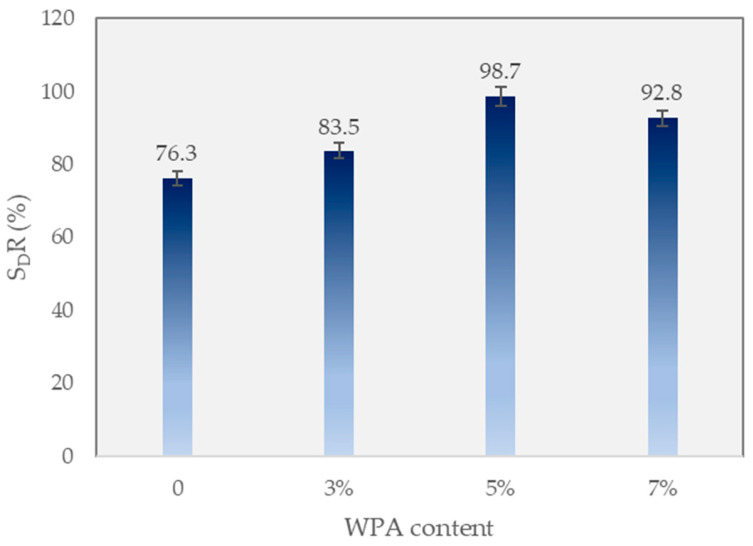
Deformation ratio.

Figure 6 provides information on the deformation strength ratio (S_D_R) of different asphalt mixtures containing WPA with varying WPA content. The control mixture, without any WPA, exhibits an S_D_R of 76.3%. However, as the WPA content increases in the WPCM mixtures, the S_D_R values show significant improvement. The WPCM mixture with 3% wt epoxy resin to asphalt binder, combined with WPA 3% wt, yields an S_D_R of 83.5%. Further increasing the WPA content to 5% and 7% wt in the WPCM mixtures results in higher S_D_R values of 98.7% and 92.8%, respectively. These findings demonstrate that incorporating WPA, along with a fixed 3% wt epoxy resin content, effectively reduces the potential for stripping damage in the asphalt mixtures, enhancing their overall performance and durability.

### 3.4. Indirect Tensile Strength (ITS)

**Figure 7:**
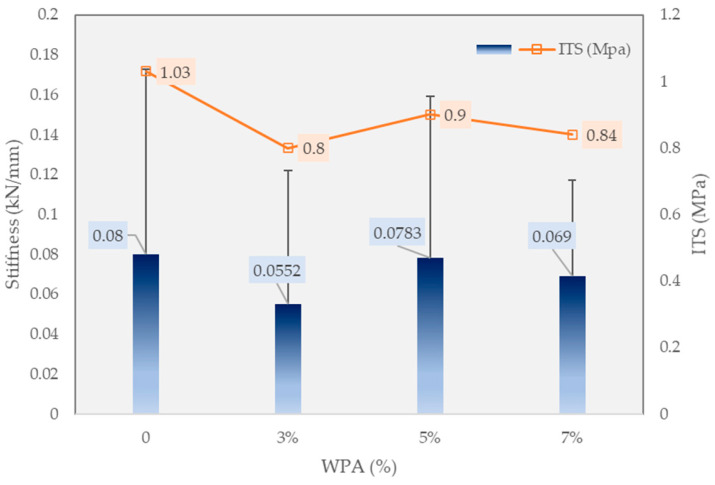
Indirect tensile strength results.

Figure 7 provides a comparative analysis of the effect of different percentages of WPA on the indirect tensile strength (ITS) and stiffness of the asphalt mixtures. The measurements are presented in megapascals (MPa) for ITS and kilonewtons per millimeter (kN/mm) for stiffness. The results demonstrate distinct changes in ITS and stiffness as the WPA content varies. The control mix with 0% wt WPA exhibits an ITS of 1.03 MPa and a stiffness of 3.2 kN/mm. With the addition of 3% wt WPA, the ITS decreases to 0.8 MPa, indicating a reduction in strength, while the stiffness decreases to 2.3 kN/mm. On the other hand, the 5% wt WPA mixture shows a slightly improved ITS of 0.9 MPa compared to the 3% wt WPA mix, while the stiffness increases to 2.7 kN/mm. The 7% wt WPA mixture demonstrates a similar ITS value of 0.84 MPa as the 3% wt WPA mix, while the stiffness remains constant at 2.3 kN/mm. These findings reveal that the inclusion of WPA in the asphalt mixture has a noticeable impact on both ITS and stiffness properties. The results indicate that a higher WPA content does not necessarily translate to improved ITS and stiffness. Rather, an optimal WPA content needs to be determined to achieve the desired balance between strength and stiffness. Such insights are valuable for designing asphalt mixtures with enhanced performance and sustainability, contributing to the development of resilient and eco-friendly infrastructure.

### 3.5. Wheel Tracking (WT) Rut Depth and Dynamic Stability

**Figure 8:**
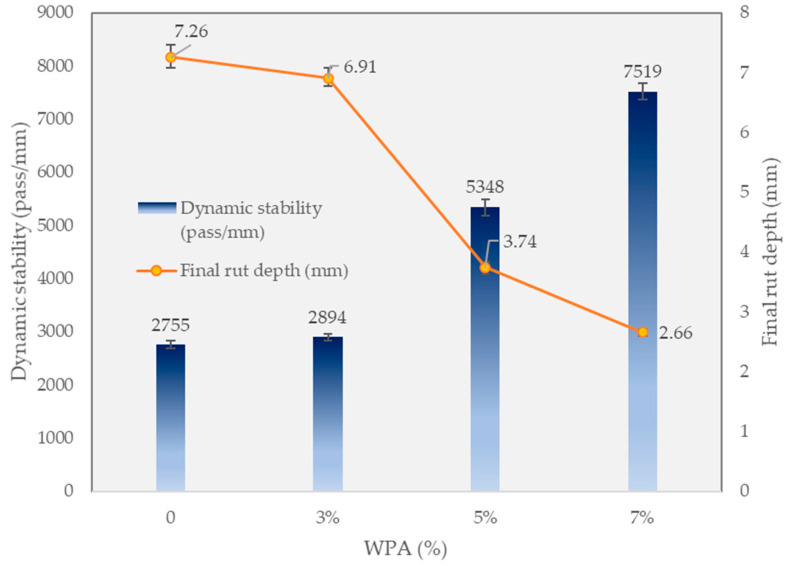
Wheel tracking test results.

Figure 8 provides a comprehensive analysis of the influence of varying percentages of WPA on the final rut depth and dynamic stability of the asphalt mixtures. It also highlights the role of epoxy resin in the WPA mix and identifies the best-performing mixture. Comparing the results, it is evident that the inclusion of WPA contributes to improved performance in terms of final rut depth and dynamic stability. The control mixture without WPA exhibits a final rut depth of 7.26 mm, indicating moderate resistance to rutting. However, as the WPA content increases, there is a significant reduction in final rut depth.

The 3% wt WPA mixture shows a decrease in final rut depth to 6.91 mm, indicating improved rutting resistance compared to the control mixture. This improvement can be attributed to the reinforcing effect of the epoxy resin added to the WPA mix, enhancing the cohesion and stability of the asphalt mixture. Further increasing the WPA content to 5% wt yields even better results, with a final rut depth of 3.74 mm. This significant reduction demonstrates the superior rutting resistance achieved by incorporating a higher percentage of WPA. The presence of epoxy resin in the WPA mix contributes to improved performance by enhancing the adhesion between the plastic particles and the asphalt binder, resulting in a more robust and durable mixture.

The 7% wt WPA mixture stands out as the best-performing mixture in terms of both final rut depth and dynamic stability. It exhibits a remarkably low final rut depth of 2.66 mm, indicating exceptional resistance to rutting. Moreover, the dynamic stability reaches 7519 passes per millimeter, emphasizing the excellent load-bearing capacity of the asphalt mixture. The epoxy resin reinforcement further enhances the cohesion and resilience of the WPA mix, contributing to its superior performance. The findings demonstrate that increasing the WPA content, coupled with the incorporation of epoxy resin, significantly improves the rutting resistance and structural integrity of the asphalt mixtures. These improvements are crucial for constructing durable and long-lasting road surfaces capable of withstanding heavy traffic loads and reducing maintenance requirements. These results highlight the potential of WPA, combined with epoxy resin, as a sustainable and effective solution for enhancing the performance and sustainability of asphalt pavements.

**Figure 9:**
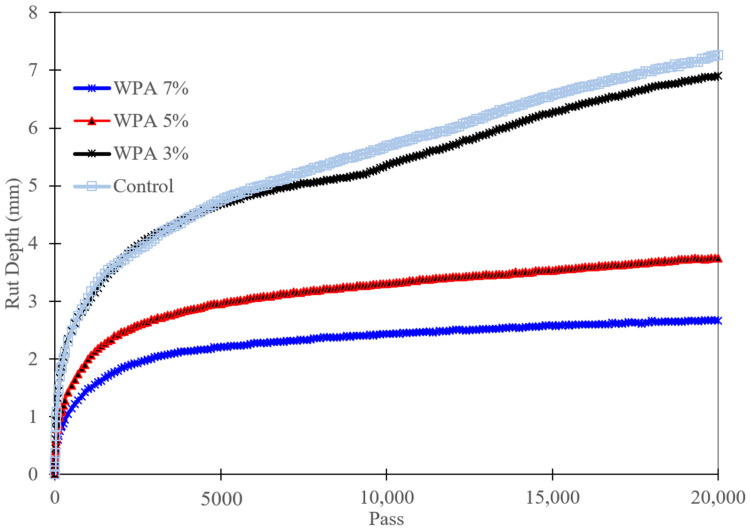
Relationship between rut depth and passing wheel.

The conclusion drawn from this research is reinforced by the results obtained from the Hamburg Wheel Tracking (HWT) graph (see Figure 9). The graph clearly illustrates the contrasting rutting behaviors between the WPA7% mixture and the control mix. While the rutting behavior of the WPA7% mixture gradually increases over time, indicating its ability to withstand deformation, the control mix exhibits a sharp increase in rutting. This observation further supports the superior rutting resistance offered by the WPA7% mixture.

Additionally, it is worth noting that none of the mixtures in the study showed any signs of stripping, highlighting the effectiveness of the asphalt mix design and the absence of moisture-related issues in the experimental asphalt specimens. The presence of epoxy resin in the WPA mixture plays a crucial role in mitigating the potential effects of stripping points, ensuring better adhesion between the asphalt binder and the aggregate.

### 3.6. Dynamic Modulus Test

**Figure 10:**
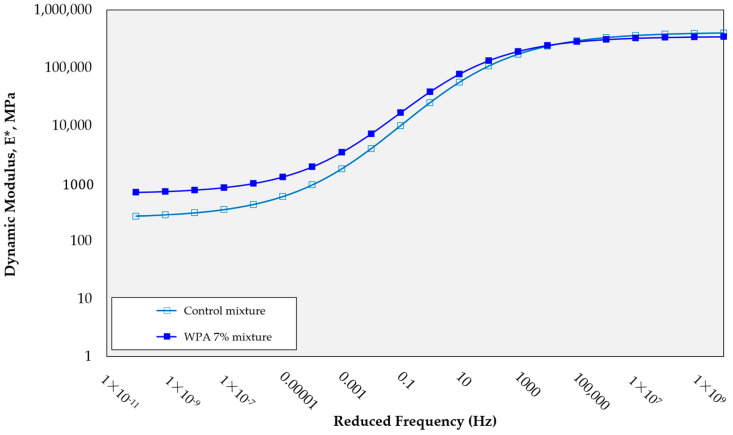
Dynamic modulus test results.

The research findings are further supported by the additional dynamic modulus test, which provides further validation of the results, as shown in Figure 10. The dynamic modulus of the WPA mixture at very low frequencies is significantly higher compared to that of the control mixture, with values of 696 and 270 MPa, respectively. This notable difference in dynamic modulus underscores the outstanding rutting resistance of the WPA mixture, especially in low-speed zones and at low frequencies. This enhanced performance can be attributed, in part, to the presence of epoxy resin in the WPA mixture. The epoxy resin acts as a reinforcing agent, improving the overall strength and stiffness of the mixture. This reinforcement contributes to the WPA mixture’s ability to withstand deformation and maintain its structural integrity, making it a promising solution for enhancing the long-term performance and durability of asphalt pavements, particularly in areas prone to rutting.

### 3.7. Discussion

The findings of this research align with previous studies and contribute to the existing knowledge on the incorporation of WPA, epoxy resin, and magnesium in HMA mixtures. The observed improvements in deformation strength, adhesion, and cohesion properties with increasing WPA content are consistent with similar investigations conducted by researchers Safeer et al. (2021) [39] and Yao et al. (2022) [16]. These studies have also reported enhanced load-bearing capacity with the proper addition of plastic aggregate. Therefore, the results obtained in this research confirm the positive influence of WPA on the mechanical properties of HMA.

Moreover, the findings regarding the benefits of incorporating epoxy resin in the HMA mixture are in line with prior research from Jing et al. (2023) [40] and Xue and Wu (2023) [21]. The increased adhesion and cohesion properties observed in the present study demonstrate the reinforcing effect of epoxy resin, which improves the bonding between the asphalt binder and aggregate particles. This enhanced bonding contributes to improved load-bearing capacity and resistance to stripping, aligning with the reported outcomes from previous studies. Consequently, the results from this research further validate the effectiveness of epoxy resin as a reinforcing agent in HMA. Additionally, the utilization of magnesium as an additive in the HMA mixture provides notable benefits consistent with previous investigations of Chen et al. (2022) [23] and Teresa et al. (2023) [41].

The observed improvements in strength underscore the positive impact of magnesium on the stiffness and viscoelastic behavior of the asphalt mixture. These findings align with prior research, which has highlighted the role of magnesium in enhancing the mechanical properties of HMA. Therefore, the results obtained in this research confirm the valuable contribution of magnesium as an additive in improving the performance of HMA.

Overall, the findings of this research align with previous studies and provide further evidence supporting the positive impact of incorporating WPA, epoxy resin, and magnesium in HMA mixtures. The observed improvements in various mechanical properties validate the effectiveness of these additives in enhancing the performance, durability, and sustainability of asphalt pavements.

## 4. Conclusions

This study examined the impact of incorporating 3%, 5%, and 7% wt of waste plastic aggregate (WPA) and 3% wt epoxy resin in the asphalt mixtures, evaluating various properties such as deformation strength, ITS, rut depth, and dynamic stability, and promoting the recycling of by-products in pavement. The following findings can be drawn from the manuscript:

- The control mixture, without any WPA, exhibited a deformation strength of 4.29 MPa. The WPCM mixture with 3% wt WPA and epoxy reinforcement had a deformation strength of 4.01 MPa. Increasing the WPA content to 5% and 7% wt resulted in deformation strengths of 3.7 MPa and 3.32 MPa, respectively. The presence of epoxy resin enhanced the bond between WPA and the asphalt binder, improving cohesion and load-bearing capacity.
- The inclusion of WPA in the asphalt mixtures affected the indirect tensile strength (ITS) and stiffness properties. The optimal WPA content for balancing strength and stiffness varied, with the 5% wt WPA mixture demonstrating a slightly improved ITS of 0.9 MPa and increased stiffness of 2.7 kN/mm compared to the 3% wt WPA mix. Higher WPA content did not necessarily lead to improved ITS and stiffness. These findings contribute to the development of sustainable asphalt mixtures with enhanced performance and resilience.
- The control mixture showed moderate rutting resistance, with a final rut depth of 7.26 mm. Increasing the WPA content resulted in significant reductions in rut depth, with the 3% wt WPA mixture achieving 6.91 mm and the 5% wt WPA mixture further reducing it to 3.74 mm. The best-performing mixture was the 7% wt WPA, exhibiting exceptional performance with a final rut depth of 2.66 mm and a dynamic stability of 7519 passes per millimeter. The addition of epoxy resin enhanced cohesion and adhesion between plastic particles and the asphalt binder, contributing to improved performance.
- The inclusion of epoxy resin in the WPA mixture also played a critical role in preventing stripping points and improving adhesion between the asphalt binder and the aggregate.
- The dynamic modulus of the WPA mixture at very low frequencies is significantly higher (696 MPa) than that of the control mixture (270 MPa). This notable difference underscores the outstanding rutting resistance of the WPA mixture, particularly in low-speed zones and at low frequencies.
- The inclusion of WPA in the asphalt mixtures, along with a fixed 3% wt epoxy resin content by weight, significantly improves the S_D_R. The control mixture exhibits an S_D_R of 76.3%, while the WPCM mixtures with 3%, 5%, and 7% wt WPA content achieve S_D_R values of 83.5%, 98.7%, and 92.8%, respectively.
- While this research provides valuable insights into the use of WPA and epoxy resin in asphalt mixtures, further field studies are necessary to assess their long-term durability. Future research should explore the effects of different types and sizes of waste plastic materials, investigate synergistic combinations with additives/modifiers, and conduct life-cycle assessments for a comprehensive understanding of their sustainability and cost-effectiveness.

## Figures and Tables

**Table 1:** Properties of PG64-22 asphalt binder used in this study.

Test Item		Specifications	PG 64-22 (Pen.60-80)
Penetration at 25 °C, dmm [24]		-	75.0
Flashpoint, °C [25]		>260	339.0
The softening point, °C [25]		-	45.8
Penetration Ratio, % [24]		>55	64.1
Rotational viscosity at 120 °C, mm^2^/s [26]		-	928
DSR	Original G*/sin δ (kPa, 64 °C) [27]	>1.0	1.41
	RTFO G*/sin δ (kPa, 64 °C) [27]	>2.2	3.28
BBR	Stiffness (MPa, −12 °C) [28]	<300	208
	M-value (−12 °C) [28]	>0.3	0.27

**Table 2:** Properties of SK epoxy resin.

Adhesive Strength (MPa)	Stiffness (MPa)	Water Absorption (%)	Glass Transition Temperature (°C)	Viscosity (cps)
4–6	2600	<0.1	52	5900

**Table 3:** Properties of natural aggregate.

Classification		Density (g/cm^3^)			Absorption (%)	Abrasion (%)
		Bulk	SSD	Apparent		
	20 mm	2.748	2.761	2.785	0.485	26.109
	13 mm	2.708	2.735	2.784	1.030	33.300
Fine Agg.	Screenings	2.721	2.776	2.880	2.060	
Filler	Limestone powder		0.000	2.793		

**Table 4:** Properties of WPA.

Waste Plastic Agg.	Year	Gradation						Fineness Modulus	Bulk The Density of Agg.	Specific Gravity under Oven Dry	Solid Content of Agg.
		25 mm	20 mm	10 mm	5 mm	2.5 mm	1.2 mm				
WPCM	1st	100	97.5	24.9	6.3	1.1	0.1	6.5	0.5	1.3	36.1

**Table 5:** Wheel tracking designation of WPCM.

Layer	Gradation	Mix by WPA	WPA Content (wt. %)			
			0	3	5	7
Base	BB-2	Control	○	-	-	-
		WPCM	-	○	○	○

**Table 7:** Mix design results.

Type	Gradation	Asphalt Content (wt. %)	Density	Air Voids (%)	VMA (%)	VFA (%)	SD (MPa)
Control	BB-2	4.0	2.389	5.4	14.8	63.4	3.03
		4.5	2.370	5.5	15.9	65.7	2.79
		5.0	2.421	2.7	14.6	81.4	4.09
		5.5	2.416	2.2	15.2	85.5	4.26
		6.0	2.419	1.4	15.6	91.3	4.24
WPCM	BB-2	4.2	2.305	4.7	14.1	67.1	2.80
		4.7	2.313	3.7	14.3	74.5	2.75
		5.2	2.317	2.9	14.6	80.5	3.23

**Table 8:** The OAC of each mix is determined from the mix design.

Gradation	OAC (%) of Each Mix	
	Control	WPCM
BB-2	4.2	4.2

## Data Availability

Not applicable.
